# Prediction of thermal conductivity of polyvinylpyrrolidone (PVP) electrospun nanocomposite fibers using artificial neural network and prey-predator algorithm

**DOI:** 10.1371/journal.pone.0183920

**Published:** 2017-09-21

**Authors:** Waseem S. Khan, Nawaf N. Hamadneh, Waqar A. Khan

**Affiliations:** 1 Department of Mechanical & Industrial Engineering, College of Engineering Majmaah University, Majmaah, Kingdom of Saudi Arabia; 2 Department of Basic Sciences, College of Science and Theoretical Studies, Saudi Electronic University, Riyadh, Kingdom of Saudi Arabia; Tsinghua University, CHINA

## Abstract

In this study, multilayer perception neural network (MLPNN) was employed to predict thermal conductivity of PVP electrospun nanocomposite fibers with multiwalled carbon nanotubes (MWCNTs) and Nickel Zinc ferrites [(Ni_0.6_Zn_0.4_) Fe_2_O_4_]. This is the second attempt on the application of MLPNN with prey predator algorithm for the prediction of thermal conductivity of PVP electrospun nanocomposite fibers. The prey predator algorithm was used to train the neural networks to find the best models. The best models have the minimal of sum squared error between the experimental testing data and the corresponding models results. The minimal error was found to be 0.0028 for MWCNTs model and 0.00199 for Ni-Zn ferrites model. The predicted artificial neural networks (ANNs) responses were analyzed statistically using z-test, correlation coefficient, and the error functions for both inclusions. The predicted ANN responses for PVP electrospun nanocomposite fibers were compared with the experimental data and were found in good agreement.

## Introduction

**Fig 1:**
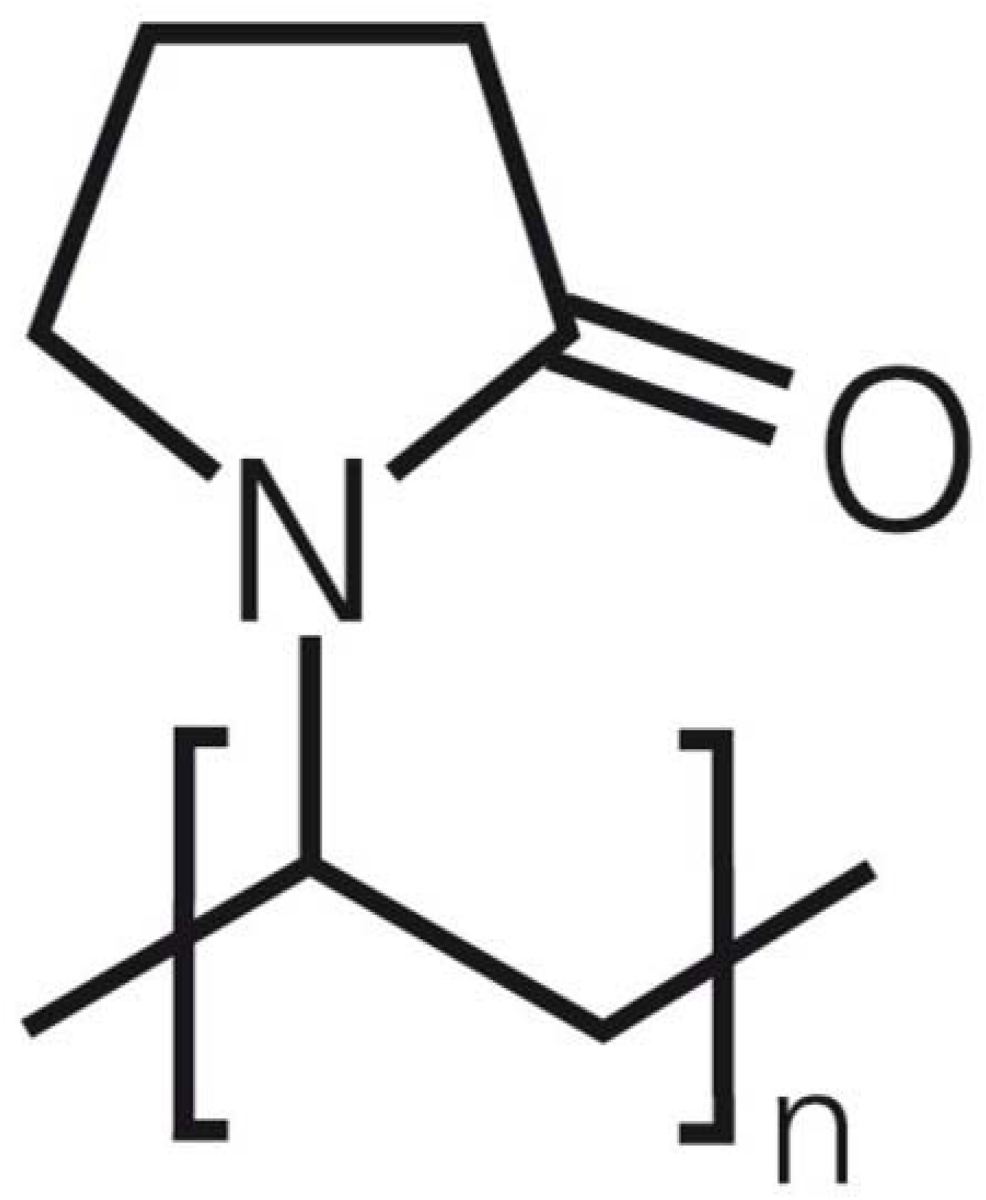
Chemical structure of PVP.

PVP is a water-soluble chemically inert amorphous polymer (-CH_2_CHC_4_H_6_NO-) _*n*_ made from the monomer *N*-vinylpyrrolidone, used chiefly in medicine as a vehicle for drugs. In dry state, it is a light flaky powder, which readily absorbs up to 40% of its weight in atmospheric water. It can be used as a binder in many pharmaceutical tablets and capsules. In solution form, it has excellent wetting properties and quickly form film, which makes it a very good additive to coatings. That is why; it is used in glue stick, inkjet papers and inks. The chemical structure of PVP is shown in Fig 1 PVP possess good adhesion, good complexation, and low toxicity, high solubility in both polar and non-polar solvents biocompatibility, good spinnability and capability to interact with many hydrophilic materials [1–3]. PVP has a wide range of industrial applications such as food, cosmetic, pharmaceutical, adhesives, paints, detergents and energy storage [1,4].

Recently, electrospinning has gained much interest in research community because not only it can generate polymeric fibers with nano diameter by employing an electrically driven jet of polymeric solution or melts, but it has the principal advantages of being a very simple and cost-effective process, compared to conventional fibers forming processes [5].

**Fig 2:**
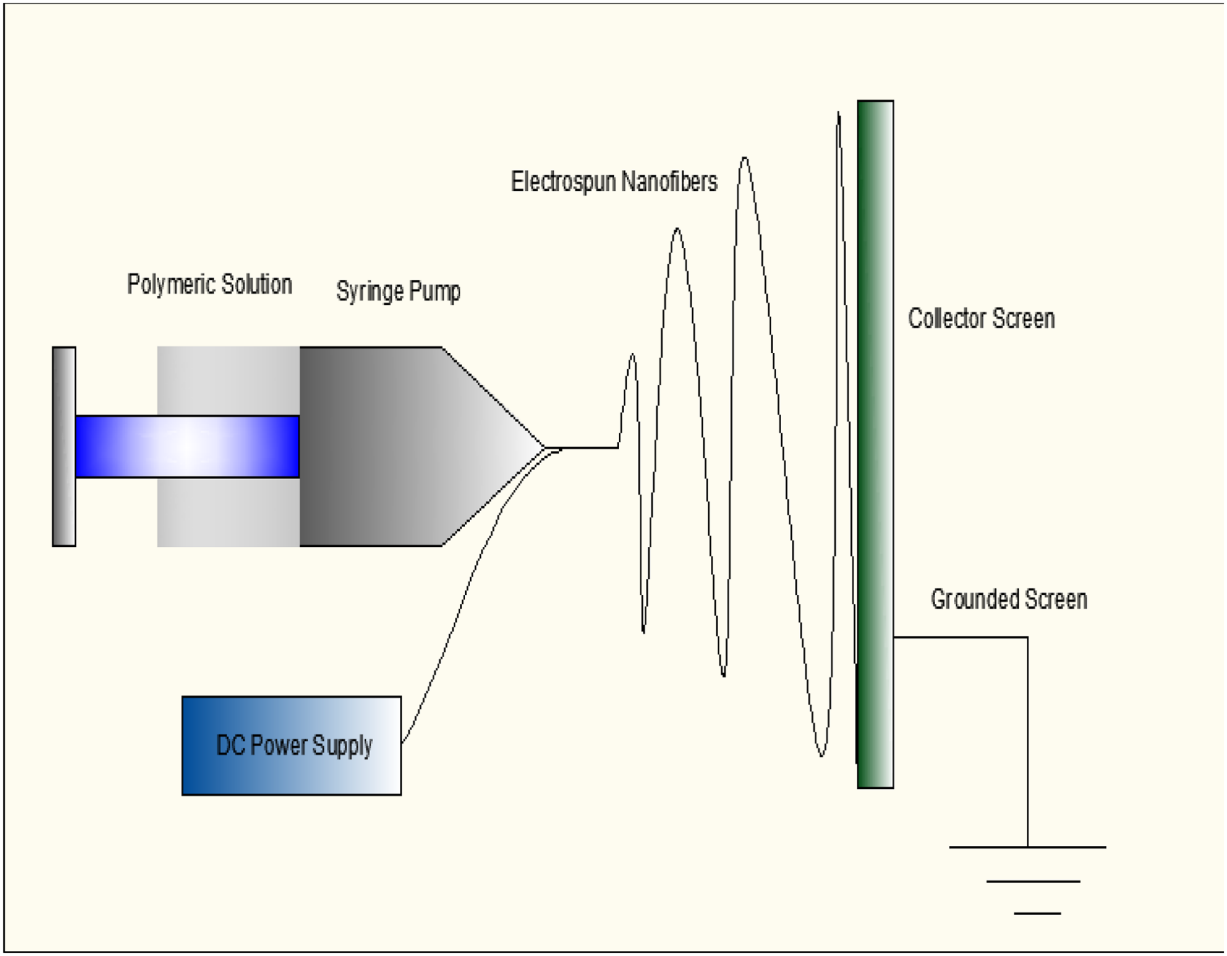
Schematic view of electrospinning process.

Electrospun nanofibers with their huge surface areas to volume ratio, about a thousand times, higher than that of a human hair, have the potential to significantly improve current technology and find various applications in new areas due to their fascinating properties [5]. Nanofibers possess unique features, such as a nanoscaled dimension in the cross-sectional area, a macroscopic length on the axis of fibers, high surface area, and a porous structure, and are generally referred to as ultrafine fibers [6]. Electrospinning is an electrostatically-driven process that produces fibers in nanometer to micrometer diameters [7]. Specifically, when a high voltage is applied to polymeric solution or melt, electrostatic repulsion overcomes surface tension, which repeatedly stretch and split longitudinally into ultrafine fibers [7,8]. If the molecular cohesion or chain entanglement in the polymeric solution is sufficiently high, the droplet would not break-up, but continue to stretch thousands of time to form very fine fibers on the collector screen, which is the primary mechanism for the generation of nanosized fibers [9,10]. Fig 2 shows a schematic illustration of an electrospinning process.

In this study, Ni-Zn ferrite and Multiwalled carbon nanotubes (MWCNTs) were incorporated in PVP matrix to fabricate electrospun nanocomposite fibers. Ni-Zn ferrite is a single-phase powder having spinel structure. Ni-Zn ferrite is a semi-conductor material. Ni-Zn ferrites have nearly three orders of magnitude lower thermal and electrical conductivity values than MWCNTs. Generally, polymers are considered as non-conductive materials due to their low conductivities; however, conductive polymeric composites can be fabricated by embedding additives/fillers, such as CNTs, C_60_, graphene, carbon blacks, as well as indium tin oxide (ITO), and other metallic and ceramic structured particles/inclusions into the polymeric matrices [11]. Ni-Zn ferrites possess high thermal conductivity due to the nature and structure of these particles. These nanoparticles have a higher number of phonons vibrational modes and higher mean free path, as well due to their crystalline nature [12]. It is well-known that the thermal conductivity of multiwalled carbon nanotubes (MWCNTs) is 3 to 4 times higher than many metals such as copper and silver. Carbon nanotubes offer unique properties such as high strength, lightweight, high thermal and air stability, elasticity, high thermal and electrical conductivities and high aspect ratio. Therefore, they possess tremendous advantages over other filler materials in composite fabrication. The outstanding properties of carbon nanotubes make them an ideal filler material for advanced nanocomposite applications.

Artificial neural networks (ANNs) are a mathematical or computational model which is constructed employing inspiration from the functional aspects of biological neural network [13–15]. In this study, a neural network approach was used to predict the thermal conductivity of PVP electrospun nanocomposite fibers as function of weight % of MWCNTs and Ni-Zn ferrites. Experiments were performed on PVP nanocomposite fibers. In developing the ANN model, several configurations were evaluated. Optimal neural network was selected with one input layer, one hidden layer and one output layer. The networks were trained using prey predator algorithm (PPA) and then tested with untrained values. PPA is a new metaheuristic algorithm, introduced by Tilahun and Ong [16]. It is inspired by the interaction between a predator and prey of animals in the ecosystem [17,18]. PPA Predicted thermal conductivity values obtained from network were examined statistically and compared with actual values obtained from experiments. Several error functions were also used to check the goodness of fit of the models.

## Experimental

### Materials

PVP (130,000g/mol) and ethanol were purchased from Sigma-Aldrich and used without any further purification. Multiwalled carbon nanotubes (MWCNTs) with a diameter of 140 (± 30) nm and a length of 7 (± 2) μm were purchased from Fisher Scientific. Ni_0.6_Zn_0.4_Fe_2_O_4_ (Ni-Zn ferrite) nanoparticles (21.5 nm) were prepared using a co-precipitation technique. In this process, Ni-, Zn-, and Fe-sulfates (NiSO_4_, ZnSO_4_, and Fe_2_ (SO_4_)_3_) were dissolved in deionized water, and heated to 80°C with constant stirring at 700 rpm for 2–3 hours. NaOH solution was added slowly in the solution in order to initiate chemical reaction. After about 2 hours of agitation under magnetic stirrer, the ferrite particles (Ni_0.6_Zn_0.4_Fe_2_O_4_) were formed and began to precipitate. After washing with DI water several times, fine ferrite (nanosize) particles were produced. The powder sample was then dried at room temperature.

### Method

**Fig 3:**
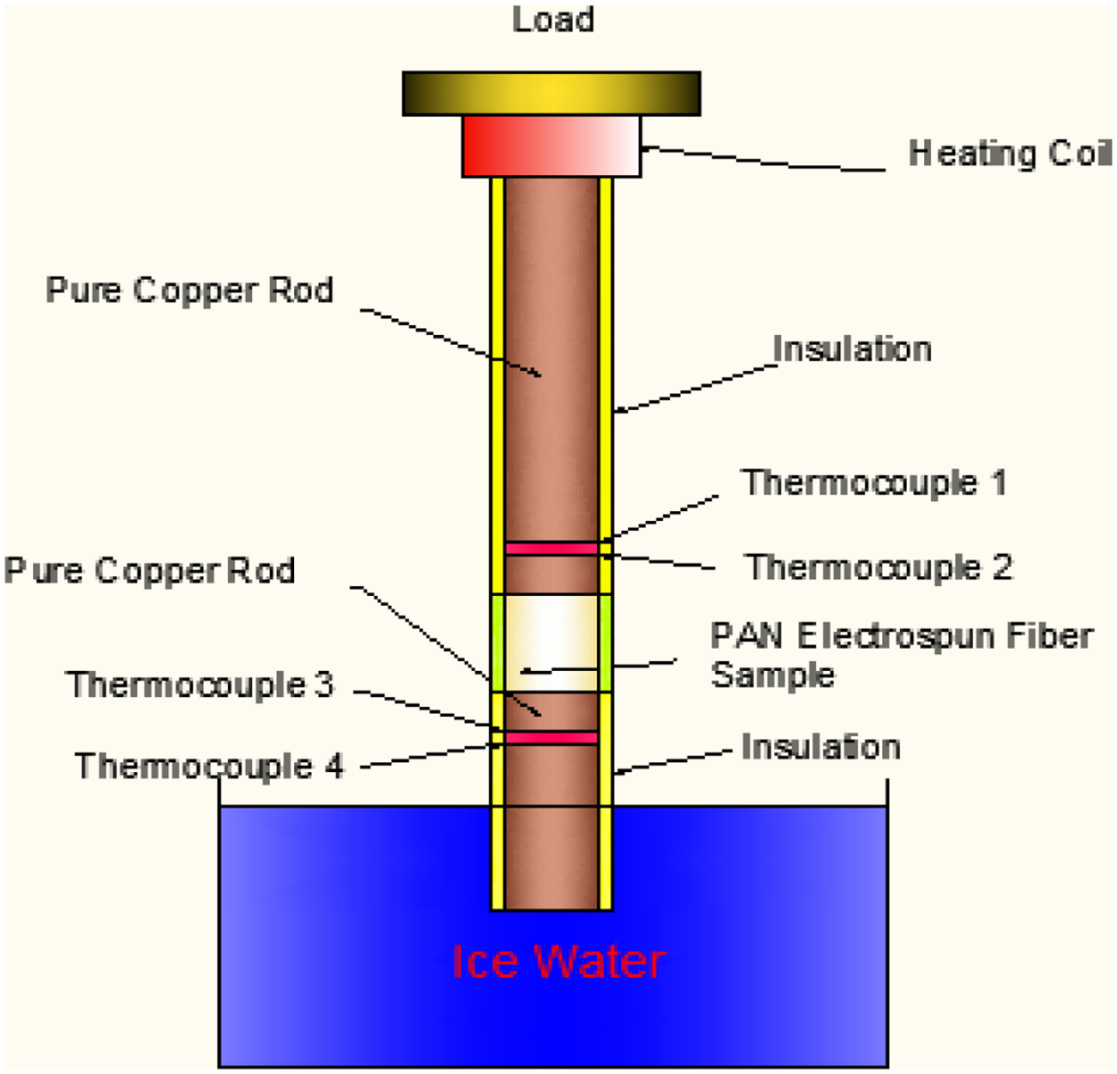
Experimental arrangement of thermal conductivity measurement.

Different wt. % of MWCNTs (0%, 1%, 2%, 4% and 8%) and Ni_0.6_Zn_0.4_Fe_2_O_4_ (0%, 1%, 2%, 4%, 8% and 16%) were separately dispersed in ethanol and sonicated for 30 minutes, and then calculated amount of PVP was added to the solution. The solution was then stirred on a hot plate at 60°C (700 rpm) overnight, to make a homogeneous blend of PVP polymeric solution. The well-dispersed solution was electrospun to produce consistent electrospun nanocomposite fibers for the thermal conductivity testing. The polymeric solutions containing different wt. % of MWCNTs and ferrite particle (Ni_0.6_Zn_0.4_Fe_2_O_4_) were transferred to a 10 ml plastic syringe connected to a capillary needle having an inside diameter of 0.5 mm. A platinum electrode around 0.25 mm diameter was provided at the syringe and connected to a High DC supply. The applied voltage, pump speed and capillary tip to collector screen distance was maintained at, 25kV, 1 ml/hr. and 25cm, respectively. The collector screen was grounded. Electrospun fibers were then collected on a grounded screen and dried in an oven at 60°C for 6 to 8 hours to remove all residual solvents. The thermal conductivities of PVP samples with different weigh percentages of MWCNTs and Ni-Zn ferrites were measured by comparative method. The experimental procedure and data, has been reported elsewhere [19]. The experimental arrangement for thermal conductivity testing is depicted in Fig 3.

### Artificial neural networks

ANNs are computational models which are constructed to employ inspiration from the functional aspects of biological neural network [20–23]. ANNs are composed of countless neurons known as processing units. These processing units are generally categorized in a series of layers such as input, hidden and output layers. Multilayer perception (MLP) is termed as architecture of ANNs [14]. Several algorithms have been available for training ANNs, prey predator algorithm was found to be the most effective algorithm to be used in this study. The purpose of this study was to study the effects of nanoinclusions such as Ni-Zn ferrite and MWCNTs on the thermal conductivity of PVP polymer using ANN modeling and statistical analysis.

**Fig 4:**
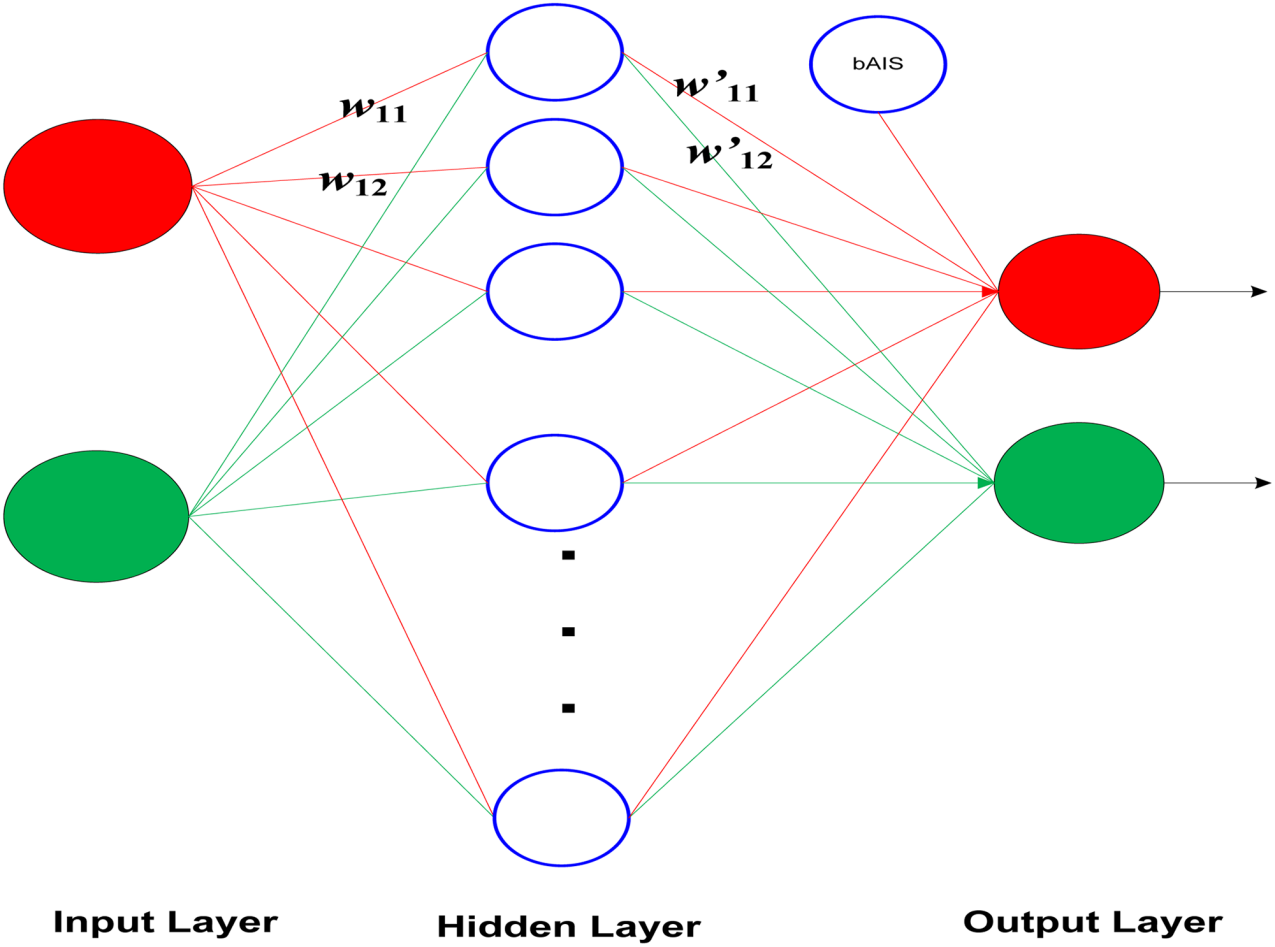
A structure of multilayer neural network.

The structure has three layers; input layer, hidden layers, and output layer. Fig 4 is a structure of the MLPNN, with one hidden layer. Each neuron possesses three unique characteristics in the network. MLPNN is termed as the architecture of ANNs. By using input vectors and corresponding output vectors, it is possible to train a network, so that it can determine a model to an arbitrary degree of accuracy.

The activation function in the hidden layer is assumed to be the sigmoid function, which can be given by Eq (1):

$$Y=\frac{1}{1+e^{x}}$$ (1)

There are two important areas in neural network, i.e. optimization and validation. In optimization, efforts are directed towards building networks that are efficient and fast. The validation means that the network need to function correctly. The adaptive neural approach was amenable to rule expression. Note that, learning in a neural network is called training. Training methods can be applied to the MLPNN parameters in order to improve the performance of the network. In this study, we have used the sum of squared error (SSE) to test the performance of the neural networks. It is given by

$$SEE=\sum {\left(Actual\;output-target\;output\right)}^{2}$$ (2)

We have used prey predator algorithm as neural learning algorithm to estimate the parameters which are the input weights and the output weights.

$$Actual\;output\;\left(yk\right)=\sum_{j=1}^{m}\sum_{i=1}^{n}w_{jk}\sum_{k=1}^{s}{w^{\prime }}_{ki}\frac{1}{1+e^{-x_{j}}}$$ (3)

where,

N: Number of hidden nouns

M: Number of input data

S: Number of input neurons

K: Number of output neurons

*w*_*jk*_: Input weight which is between the input neuron *j* and hidden neuron *k*

*w′*_*ki*_: Output weight which is between the hidden neuron *k* and hidden neuron *i*

### Prey predator algorithm

**Fig 5:**
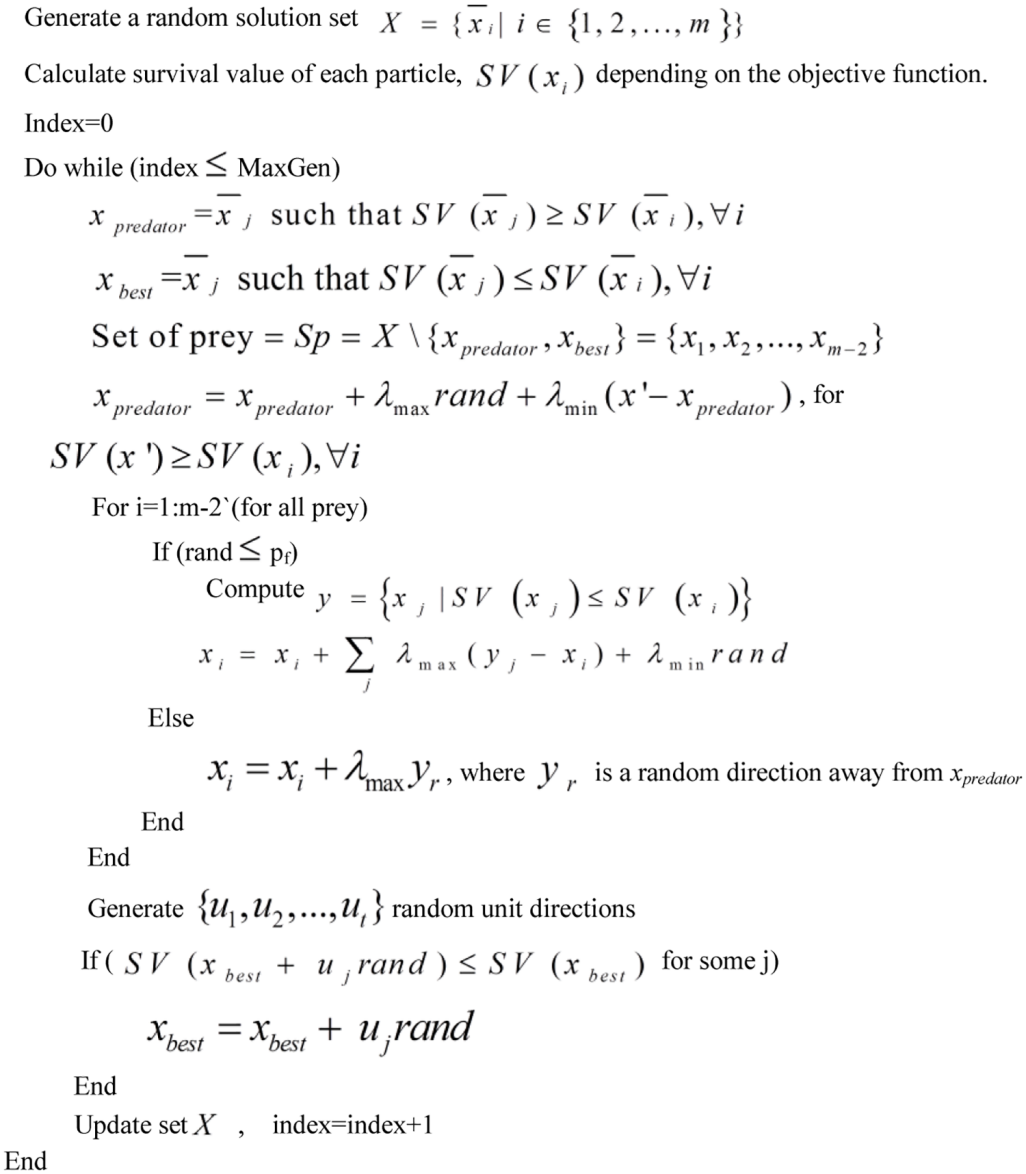
Pseudo code of PPA for a minimization problem.

Prey predator algorithm is one of the new metaheuristic algorithms for optimization problems [17]. It has better exploration properties compared to other algorithms, such as particle swarm optimization algorithm and genetic algorithm. It is inspired by the interaction between a predator and preys of animals in the ecosystem. Randomly generated solutions were assigned as a predator and preys depending on their performance on the objective function. A solution with least performance will be assigned as a predator and the others preys. A prey with better performance in the objective function will be called best prey. After the assignment of predator and preys, the preys will run away from the predator and follow preys with better performance. The predator does the exploration by running randomly and chasing the prey with least performance. The best prey in the other hand does only a local search for exploitation purpose. The main steps of the prey predictor algorithm for training experimental data are as follows (see Fig 5) [16, 17].

i. Generate random solutions.
ii. Calculate the performance of the solutions in the objective function and assign the solution with least performance as a predator, the solution with best performance as best prey and the rest as preys.
iii. Move the predator randomly towards the prey with least performance.
iv. Generate random directions around the best prey and if there is any direction which increases the performance of the best prey, move the best prey in that direction, otherwise keep the best prey in its current position.
v. If probability of follow-up is met, then move the preys towards better preys and also with a local search. If probability of follow-up is not met, then move the preys randomly away from the predator.
vi. Update the list of preys, best prey and predator.
vii. If a termination criterion is met, then stop else go to step (iii).

## Statistical study

### Hypothesis testing

The effects of nanoinclusions on the thermal conductivity of PVP polymers can be examined by applying statistical hypotheses using z-test and one-way ANOVA. It is assumed that the nanoinclusions have no effect on the thermal conductivity of PVP polymers which is equivalent to test $H_{0}:\bar{D}=0$. The alternate hypothesis *Ha*:*μ*_1_ > *μ*_2_ will confirm that the inclusions are effective and increase the thermal conductivity of PVP polymers. The results infer that, in each case, *z* > *z*_*crit*_ *and F* > *F*_*crit*_, hence the null hypothesis can be rejected and concluded that the inclusion of MWCNTs and Ni-Zn ferrites is effective and increases the thermal conductivity of PVP polymers. Furthermore, *p* < 0.05 indicates that the null hypothesis must be rejected. T-test (Eq 4) is also one of most common statistical tests for testing data [24].

$$t=\frac{r}{\sqrt{\frac{1-r^{2}}{n-2}}}$$ (4)

### Error functions

In addition to the sum of squared error function, which is mentioned in Section 3, the following error functions can be used to determine the best fit between experimental and ANN predicted results.

1. The average relative error function (ARE)
  This error function minimizes the fractional error distribution across the entire concentration range [25]. It is given by:
  $$ARE=\frac{100}{n}\Sigma \left|\frac{k_{\text{exp }t}-k_{pred}}{k_{\text{exp }t}}\right|$$ (5)
  Where *k*_exp *t*_ is the experimentally measured thermal conductivity and *k*_*pred*_ is the thermal conductivity predicted by ANN.
2. The sum of the absolute errors (EABS)
  This approach is similar to the *“ERRSQ”* function and it gives a better fit as the magnitude of the errors increase. It is given by [26]:
  $$EABS=\sum \left|k_{\text{exp }t}-k_{pred}\right|$$ (6)
3. Nonlinear Chi-square test
  This statistical tool is necessary for the best fit of experimental data. The value of chi-square is given by [24,26]:
  $$\chi^{2}=\sum \frac{{\left(k_{\text{exp }t}-k_{pred}\right)}^{2}}{k_{\text{exp }t}}$$ (7)
4. The hybrid fractional error function (HYBRID)
  This function is used to improve ERRSQ fit at low wt. % nanoinclusion concentrations. It considers both *the number of data points* and the number of parameters. The expression of this error function is given by [20]:
  $$HYBRID=\frac{100}{n-p}\sum \frac{{\left(k_{\text{exp }t}-k_{pred}\right)}^{2}}{k_{\text{exp }t}}$$ (8)

## Results and discussion

In this study, the thermal conductivities of PVP Polymer with different weigh percentages of MWCNTs and Ni-Zn ferrite were predicted using the neural networks and prey predator algorithm. The architecture of the neural networks which we used is two input neurons- one hidden layer with several neurons, and two output neurons. The trial and error method was employed to select the best number of the hidden neurons to improve the performance of the network. For that, we used the prey predator algorithm in the training process to determine the input weights and output weights to minimize the error of the neural networks. The considered PPA applied in this study has 100 local search directions, with 120 solutions and 9 predators. The MATLAB software has been used to implement the PPA and ANNs. Once trained, the neural network predicts the output values from given input values, and therefore acts as a “prediction model”. To train the neural network, we used 60% data as training data. PPA is executed 50 times with 500 iterations in order to find best model that has minimum error. As mentioned above, the error is the difference between the actual values and the predicted values in training data.

**Fig 6:**
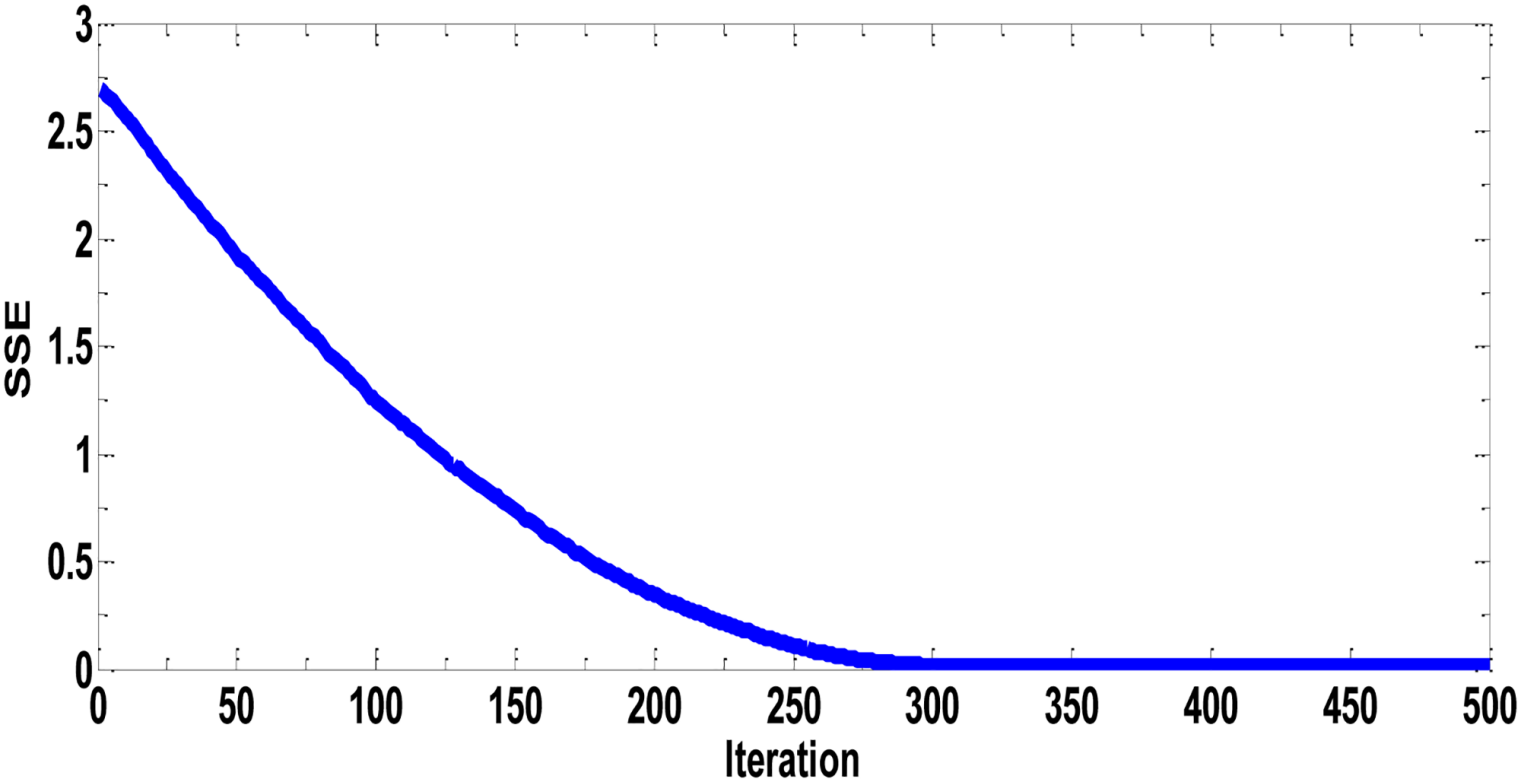
Best performance of prey predator algorithm in terms of sum of squared error for PVP with different wt. % (MWCNTs) SSE = 0.0029.

**Fig 7:**
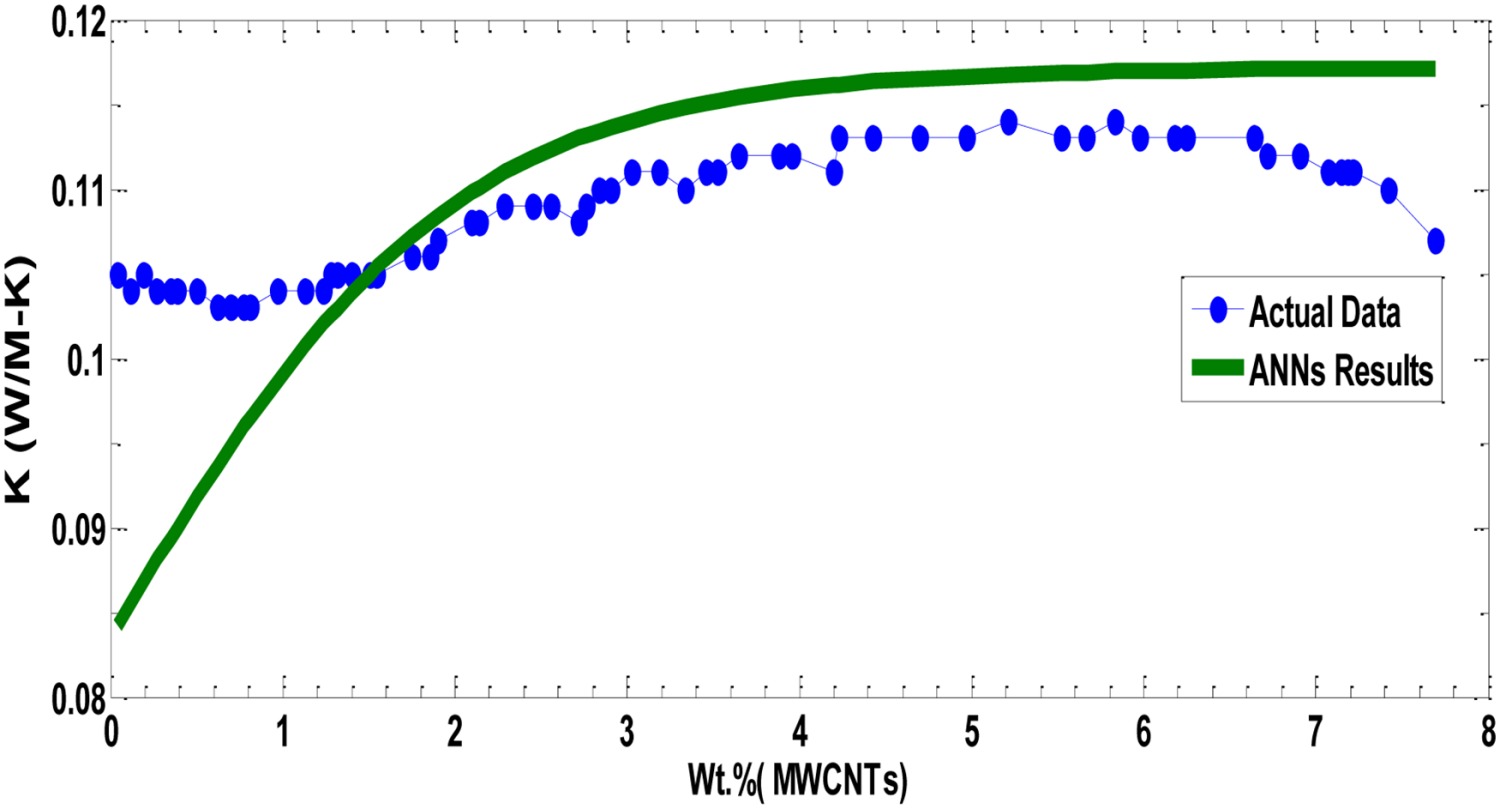
All values of PVP polymer data (wt. % of MWCNTs) with the corresponding predicted model values.

In the first experiment, we trained the neural network with PVP Polymer data (wt. % of MWCNTs was used as input data and thermal conductivity as output data). The best convergence speed of PPA in terms of SSE in MLPNN is depicted in Fig 6 with SSE = 0.0029. Fig 7 shows the thermal conductivity of PVP fibers (actual and ANN data) incorporated with different wt. % of MWCNTs. In these experiments, 0, 2, 4 and 8wt. % of MWCNTs were added in the PVP polymeric matrices prior to the electrospinning process. MWCNTs have excellent thermal (1500–3000 W/m.°K) and electrical (10^4^ S/cm) conductivity values; however, most of the polymers are thermally and electrically insulators [19,22,27]. The experimental results indicated that the addition of MWCNTs to the polymer matrices showed a significant increase in the thermal conductivity values of PVP nanocomposite fibers. The test results revealed that the thermal conductivity was increased from 0.105 to 0.12 W/m°K when the concentration was increased from 0% to 8% of MWCNTs. After the 8% MWCNTs in PVP, the electrospinning process was drastically deteriorated, due to high viscosity of PVP polymeric solution. As is seen, the overall thermal conductivities of the nanocomposite fibers are still at lower level. The reason for this low level of increase in thermal conductivity may be the interfacial resistance among MWCNTS, air packets in the fiber film, and interfacial resistance between polymeric chains and the surfaces of MWCNTs. The literature reviews showed that the thermal conductivity of MWCNTs/composite fibers depends upon the characteristic of nanotubes, their alignment, and dispersion techniques. The other possible reason for this low increase in the thermal conductivity is the amorphous nature of PVP polymer. The crystalline polymers generally have high thermal conductivity than amorphous polymers.

To examine the untrained experimental PVP Polymer data (wt. % of MWCNTs) (testing data) with the corresponding predicted model values, several statistical tests and error functions are available. The linear correlation coefficient R is a numerical measure of the strength of the relationship between two variables representing quantitative data. We used the linear correlation coefficient to test the relation between testing data with the corresponding of the ANN’ model results. The *p*-value (or probability value) is the probability of getting a value of the test statistic that is at least as extreme as the one representing the sample data, if the claim is true (whether there is correlation coefficient R). If the computed *p*-value is less than or equal to the significance level, it concludes that there is a linear correlation. Otherwise, there is no sufficient evidence to support the conclusion of a linear correlation. In addition, **|**R**|** > critical value = 0.811, concludes that there is sufficient evidence to support the claim of a linear correlation.

**Fig 8:**
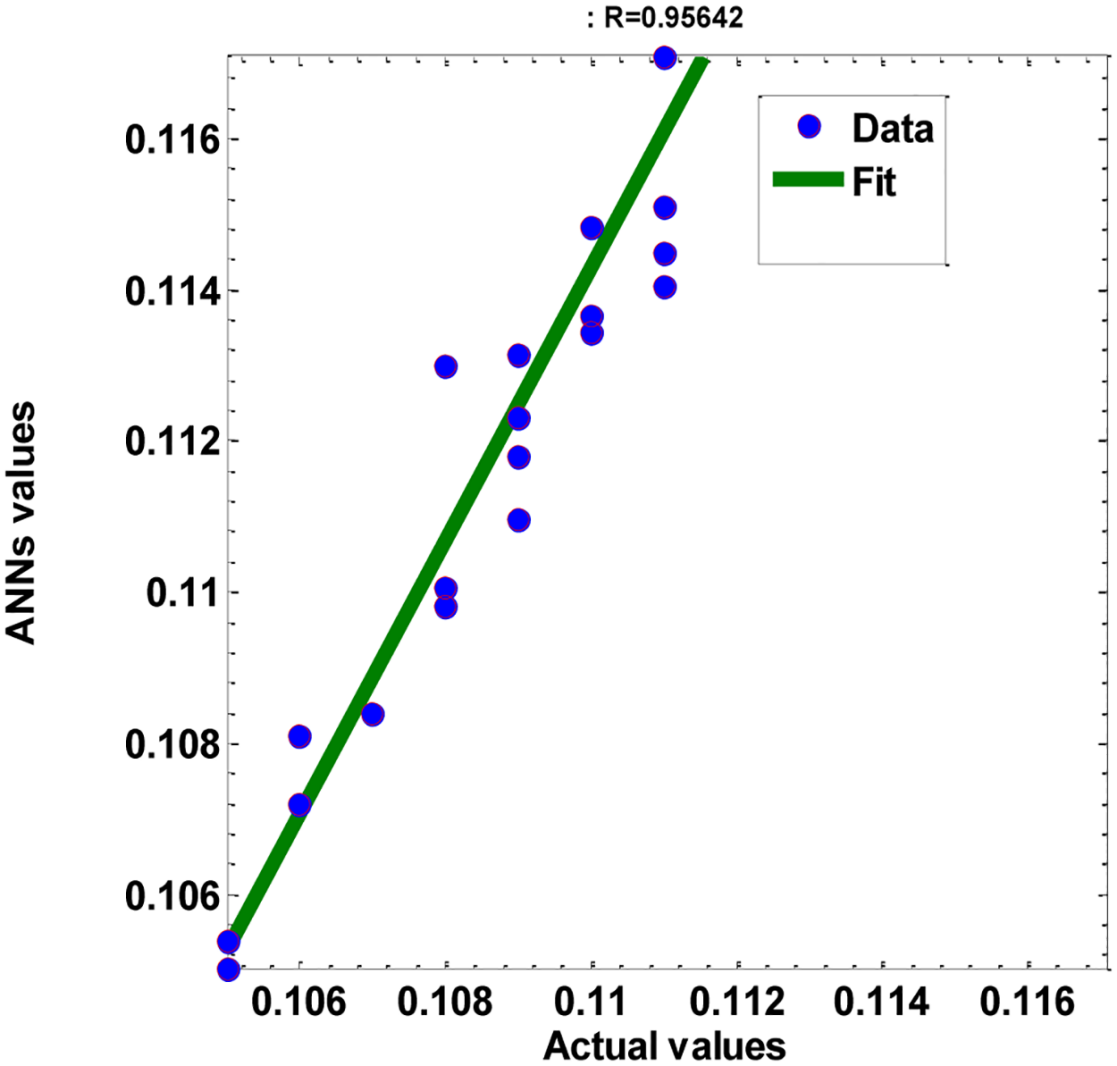
scatter plot of the testing data with the corresponding of the ANN’ model results, with (R = 0.96, and SSE = 4.68E-04).

With correlation coefficient R = 0.96 < critical value of R = 0.811, see Fig 8, n = 25 (testing samples size), *α* = 0.05 (significance level), we found that the t-value = 16.44, degree of freedom = 23, and then p-value is less than 0.00001[24]. So, the *p*-value is less than the significance level of 0.05. Accordingly, we conclude that there is sufficient evidence to support the claim of a linear correlation between the testing data and the corresponding of the ANN’ model results. In addition, the sum of squared error of testing samples with the predicted values is 4.68E-04. Accordingly, the ANN’s predictions can be used successfully to represent the PVP Polymer data with different wt. % (MWCNTs).

**Fig 9:**
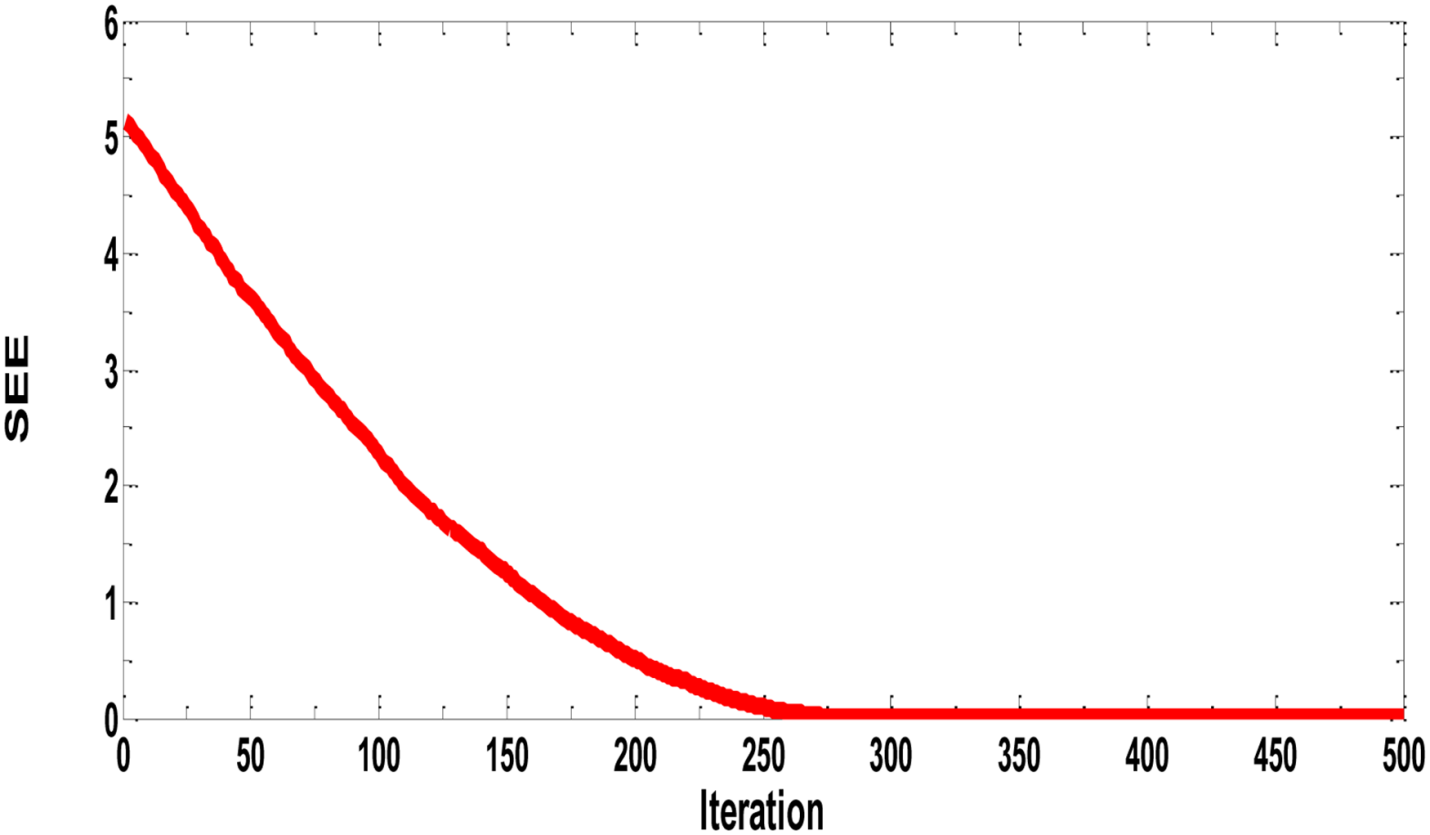
Best performance of prey predator algorithm in terms of sum of squared error for PVP with different Wt. % (NiZn Ferrites) SSE = 0.00199.

**Fig 10:**
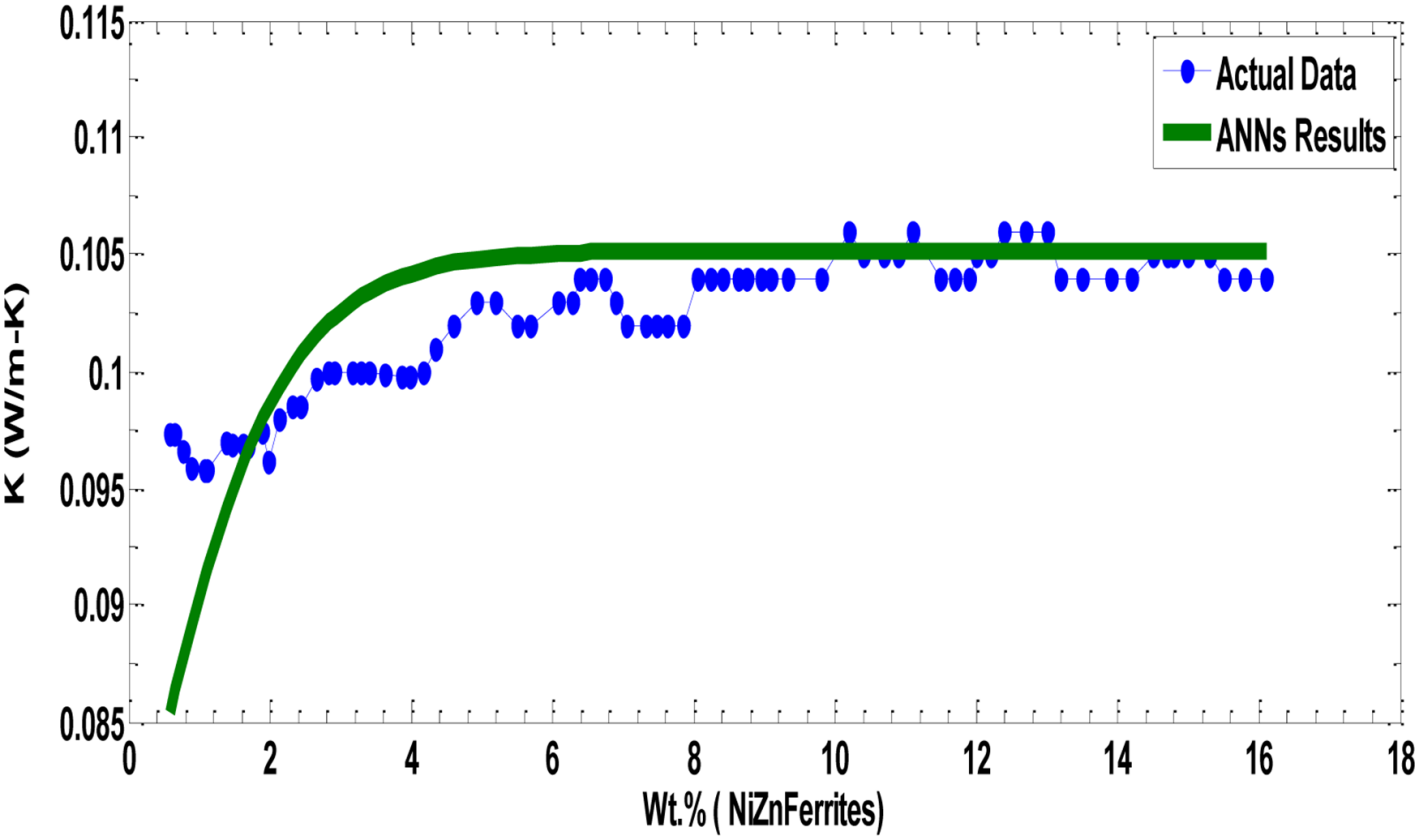
Experimental values of PVP polymer data Wt. % (NiZn Ferrites) with the corresponding predicted model values.

We repeated the same experiment with PVP data having different wt. % of Ni-Zn ferrites as input data and the thermal conductivity as output data and found the best model with minimum error, with the minimal SSE = 0.00199 (Fig 9). We found that the best model has 2 input neurons-8 hidden neurons- 2 output neurons. Fig 10 presents all experimental values of PVP Polymer data Wt. % (Ni-Zn Ferrites) with the corresponding predicted model values. The parameters of the best models in both cases are listed in Table 1.

**Table 1:** The model’ parameters for PVP with different wt. % of MWCNTs and wt. % of Ni-Zn ferrites.

Model parameters	PVP with MWCNTs	Model parameters	PVP with MWCNTs
*w*′_11_	3.21E-02	*w*_11_	7.86E-02
*w*′_21_	1.88E-01	*w*_12_	5.17E-01
*w*′_31_	6.56E-01	*w*_13_	1.24E-01
*w*′_41_	-1.03E-01	*w*_14_	6.51E-01
*w*′_51_	7.29E-01	*w*_15_	-1.40E-01
*w*′_61_	4.03E-01	*w*_16_	1.38E-01
Bais	0	*w*_bais	0

Fig 10 shows the thermal conductivities of PVP fibers incorporated with different wt. % of Ni-Zn ferrite nanoparticles. No significant improvement in thermal conductivity of PVP nanocomposite fibers was noticed. Initially, it was expected that the filling of polymer PVP with ferrites would considerably enhance their thermal conductivity, but the results revealed that the thermal conductivity was increased slightly compared to the MWCNTs based fibers. Ni-Zn Ferrite has nearly three orders of magnitude lower thermal and electrical conductivity values than MWCNTs, which could be the reason of having lower thermal conductivity in PVP nanocomposite fibers. The thermal conductivity of semi-crystalline polymer increases with crystallinity. PVP is an amorphous polymer and in amorphous polymer the mean free path is very small due to the phonon scattering caused by a number of defects in the amorphous structure, resulting in low values of a thermal conductivity [23,27–30].

**Fig 11:**
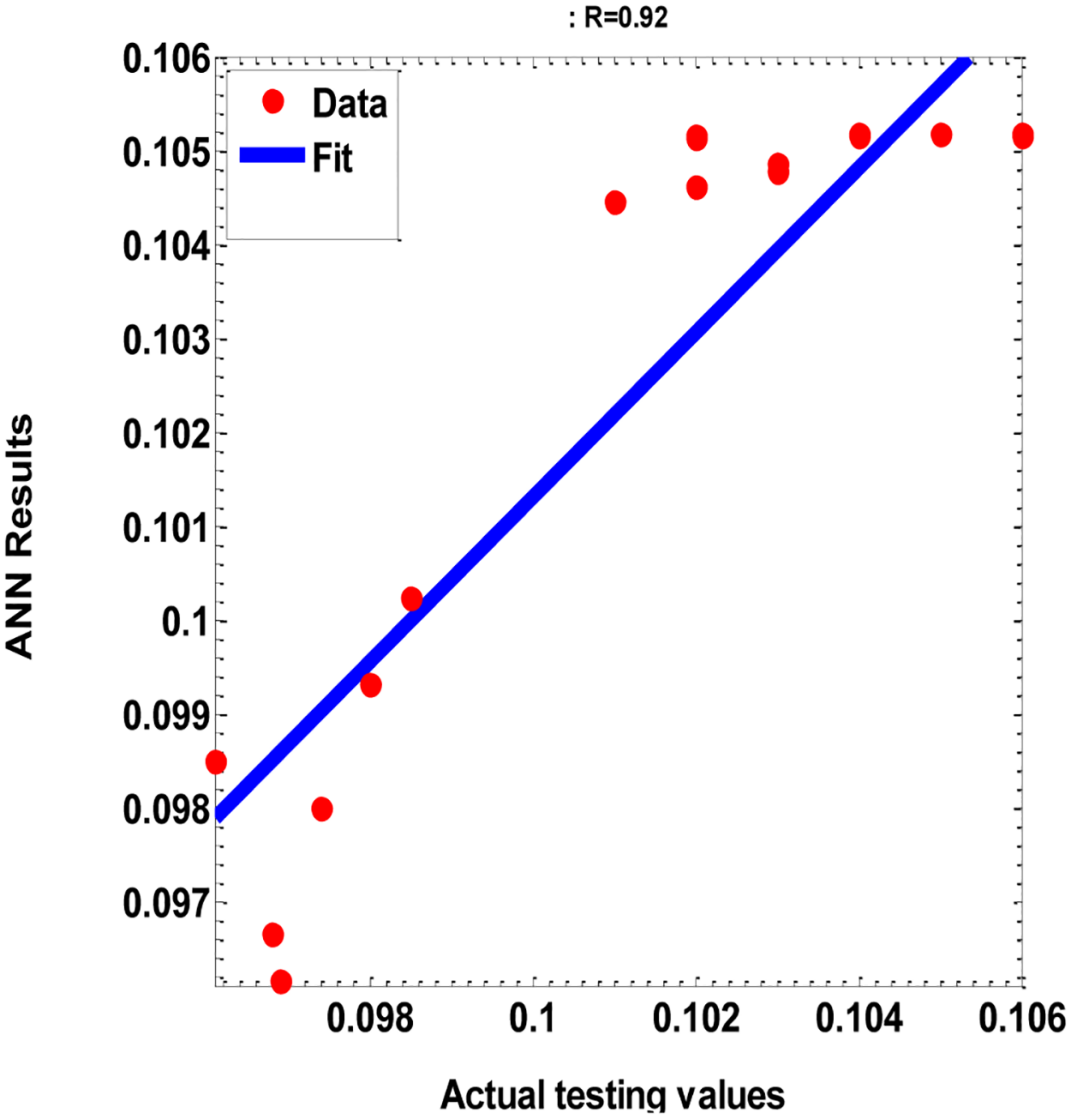
scatter plot of the testing data with the corresponding of the ANN’ model results, with (R = 0.92, and SSE = 7.19E-05).

With R = 0.92 < critical value of R = 0.811 (Fig 11), n = 28 (testing samples size), *α* = 0.05 (significance level), we found that the t-value = 11.969, degree of freedom = 26, and then p-values is less than 0.00001. So, the *p*-value is less than the significance level of 0.05.

Accordingly, we conclude that there is sufficient evidence to support the claim of a linear correlation between the testing data and the corresponding of the ANN’ model results. Moreover, the ANN model is used successfully to represent the PVP with different Wt. % (Ni-Zn Ferrites).

The results of the error functions are reported in Table 2 for each nanoinclusion. The comparison of error function reveals that ANN predictions are much better for Ni-Zn Ferrites than MWCNTs. In addition, the results of z-test and one-way ANOVA are reported in Tables 3 and 4 respectively for both nanoinclusions. The results infer that, in each case, *z* > *z*_*crit*_ *and F* > *F*_*crit*_, hence the null hypothesis can be rejected and concluded that the inclusion of MWCNTs and Ni-Zn ferrites is effective and increases the thermal conductivity of PVP polymers. Furthermore, *p* < 0.05 indicates that there is a linear correlation.

**Table 2:** Results of error functions for each inclusion.

Error Functions	PVP Polymers	
	MWCNTs	Ni-Zn Ferrites
**ARE**	3.75E-01	8.14E-01
**EABS**	0.01881	0.03849
**Chi-square**	1.12E-04	3.74E-04
**HYBRID**	2.48E-04	6.14E-04

**Table 3:** Results of z-test.

	wt%. MWCNTs	Experimental *k*	wt%. Ni-Zn Ferrites	Experimental *k*
**Mean**	3.698	0.108507246	7.212	0.102
**Variance**	6.759	1.23124E-05	23	9.03E-06
**Observations**	69	69	79	79
**Hypothesized Mean Difference**	0	0	0	0
**Z-value**	11.468		13.178	
**P(Z< = z) one-tail**	0		0	
**Z Critical one-tail**	1.645		1.645	
**P(Z< = z) two-tail**	0		0	
**Z Critical two-tail**	1.960		1.960	

**Table 4:** ANOVA results for both nanoinclusions.

Source of Variation	PVP Polymers	
	MWCNTs	Ni-Zn Ferrites
**F**	131.53	173.54
***p-value***	1.01E-21	4.06E-27
***F***_***crit***_	3.91	3.90

## Conclusions

Prey predator algorithm was used to build neural networks models. The best models have the minimal errors. The neural networks’ models are non-liner regression models. Multilayer perception neural network (MLPNN) models was employed successfully to predict the thermal conductivity of PVP Polymer with MWCNTs and Ni-Zn ferrites and the predictions have been validated statistically using statistic tests and error functions. Nonlinear regression analysis was used to minimize the error distribution between the experimental data and the neural networks data. The computed *p*-value is less than the significance level, and the computed correlation coefficients > critical value = 0.811; conclude that there is a strong linear correlation between the experimental data and the neural networks data. The increase in the thermal conductivity of PVP polymer incorporated with MWCNTs or Ni-Zn ferrites was not found as per our expectations. The predicted ANN responses for PVP polymer were compared with the experimental data and were found in good agreement.

## References

[1] Zhang D, Karki AB, Rutman D, Young DP, Wang A (2009) Electrospun polyacrylonitrile nanocomposite fibers reinforced with Fe 3 O 4 nanoparticles: fabrication and property analysis. Polymer 50: 4189–4198.

[2] Slade PE, Jenkins LT (1970) Thermal characterization techniques: Marcel Dekker.

[3] Hansen D, Ho CC (1965) Thermal conductivity of high polymers. Journal of Polymer Science Part A: General Papers 3: 659–670.

[4] Ajayan P, Stephan O, Colliex C, Trauth D (1994) Aligned carbon nanotube arrays formed by cutting a polymer resin-nanotube composite. Science-AAAS-Weekly Paper Edition 265: 1212–1214. 10.1126/science.265.5176.1212 17787587

[5] Harris PJ (2004) Carbon nanotubes and related structures: new materials for the twenty-first century. AAPT.

[6] O’connell MJ (2006) Carbon nanotubes: properties and applications: CRC press.

[7] Moniruzzaman M, Winey KI (2006) Polymer nanocomposites containing carbon nanotubes. Macromolecules 39: 5194–5205.

[8] Norkhairunnisa M, Azizan A, Mariatti M, Ismail H, Sim L (2012) Thermal stability and electrical behavior of polydimethylsiloxane nanocomposites with carbon nanotubes and carbon black fillers. Journal of Composite Materials 46: 903–910.

[9] Flaifel MH, Ahmad SH, Hassan A, Bahri S, Mou’ad AT (2013) Thermal conductivity and dynamic mechanical analysis of NiZn ferrite nanoparticles filled thermoplastic natural rubber nanocomposite. Composites Part B: Engineering 52: 334–339.

[10] Gojny FH, Wichmann MH, Fiedler B, Kinloch IA, Bauhofer W (2006) Evaluation and identification of electrical and thermal conduction mechanisms in carbon nanotube/epoxy composites. Polymer 47: 2036–2045.

[11] Balandin AA, Ghosh S, Bao W, Calizo I, Teweldebrhan D (2008) Superior thermal conductivity of single-layer graphene. Nano letters 8: 902–907. doi: 10.1021/nl0731872 18284217 10.1021/nl0731872

[12] Donadio D (2016) Simulation of dimensionality effects in thermal transport Thermal Transport in Low Dimensions: Springer pp. 275–304.

[13] Hamadneh N, Sathasivam S, Tilahun SL, Choon OH (2012) Learning logic programming in radial basis function network via genetic algorithm. Journal of Applied Sciences 12: 840.

[14] Karimzadeh F, Ebnonnasir A, Foroughi A (2006) Artificial neural network modeling for evaluating of epitaxial growth of Ti6Al4V weldment. Materials Science and Engineering: A 432: 184–190.

[15] Yeung DS, Li J-C, Ng WW, Chan PP (2016) MLPNN training via a multiobjective optimization of training error and stochastic sensitivity. IEEE transactions on neural networks and learning systems 27: 978–992. doi: 10.1109/TNNLS.2015.2431251 26054075 10.1109/TNNLS.2015.2431251

[16] Tilahun SL, Ong HC (2015) Prey-predator algorithm: a new metaheuristic algorithm for optimization problems. International Journal of Information Technology & Decision Making 14: 1331–1352.

[17] Tilahun SL, Goshu NN, Ngnotchouye JMT (2016) Prey Predator Algorithm for Travelling Salesman Problem: Application on the Ethiopian Tourism Sites Handbook of Research on Holistic Optimization Techniques in the Hospitality, Tourism, and Travel Industry: 400.

[18] Hamadneh N, Tilahun SL, Sathasivam S, Choon OH (2013) Prey-predator algorithm as a new optimization technique using in radial basis function neural networks. Research Journal of Applied Sciences 8: 383–387.

[19] Khan WS, Asmatulu R, Ahmed I, Ravigururajan TS (2013) Thermal conductivities of electrospun PAN and PVP nanocomposite fibers incorporated with MWCNTs and NiZn ferrite nanoparticles. International Journal of Thermal Sciences 71: 74–79.

[20] Porter J, McKay G, Choy K (1999) The prediction of sorption from a binary mixture of acidic dyes using single-and mixed-isotherm variants of the ideal adsorbed solute theory. Chemical Engineering Science 54: 5863–5885.

[21] Khan W, Asmatulu R, Eltabey M (2010) Dielectric Properties of Electrospun PVP and PAN Nanocomposite Fibers at Various Temperatures. Journal of Nanotechnology in Engineering and Medicine 1: 041017.

[22] Khan WS, Hamadneh N, Khan WA (2016) Polymer nanocomposites–synthesis techniques, classification and properties Science and applications of Tailored Nanostructures: One Central Press (OCP).

[23] Yadoji P, Peelamedu R, Agrawal D, Roy R (2003) Microwave sintering of Ni–Zn ferrites: comparison with conventional sintering. Materials Science and Engineering: B 98: 269–278.

[24] Triola MF (2014) Elementary Statistics Using the TI-83/84 Plus Calculator: Pearson Higher Ed.

[25] Kapoor A, Yang R (1989) Correlation of equilibrium adsorption data of condensible vapours on porous adsorbents. Gas Separation & Purification 3: 187–192.

[26] Chan L, Cheung W, Allen S, McKay G (2012) Error analysis of adsorption isotherm models for acid dyes onto bamboo derived activated carbon. Chinese Journal of Chemical Engineering 20: 535–542.

[27] Caizer C, Stefanescu M, Muntean C, Hrianca I (2001) Studies and magnetic properties of Ni-Zn ferrite synthesized from the glyoxilates complex combination. Journal of Optoelectronics and Advanced Materials 3: 919–924.

[28] Khan W, Asmatulu R, Lin Y, Chen Y, Ho J (2012) Electrospun Polyvinylpyrrolidone-Based Nanocomposite Fibers Containing (Ni. Journal of Nanotechnology 2012.

[29] Khanlou HM, Sadollah A, Ang BC, Kim JH, Talebian S (2014) Prediction and optimization of electrospinning parameters for polymethyl methacrylate nanofiber fabrication using response surface methodology and artificial neural networks. Neural Computing and Applications 25: 767–777.

[30] Giri Dev VR, Venugopal JR, Senthilkumar M, Gupta D, Ramakrishna S (2009) Prediction of water retention capacity of hydrolysed electrospun polyacrylonitrile fibers using statistical model and artificial neural network. Journal of Applied Polymer Science 113: 3397–3404.

